# Mini-Review Discussing the Reliability and Efficiency of COVID-19 Vaccines

**DOI:** 10.3390/diagnostics11040579

**Published:** 2021-03-24

**Authors:** Bogdan Doroftei, Alin Ciobica, Ovidiu-Dumitru Ilie, Radu Maftei, Ciprian Ilea

**Affiliations:** 1 Faculty of Medicine, University of Medicine and Pharmacy “Grigore T. Popa”, University Street, no 16, 700115 Iasi, Romania; bogdandoroftei@gmail.com (B.D.); dr.radu.maftei@gmail.com (R.M.); cilea1979@yahoo.com (C.I.); 2 Clinical Hospital of Obstetrics and Gynecology “Cuza Voda”, Cuza Voda Street, no 34, 700038 Iasi, Romania; 3 Origyn Fertility Center, Palace Street, no 3C, 700032 Iasi, Romania; 4 Center of Biomedical Research, Romanian Academy, Iasi Branch, Carol I Avenue, no. 8, 700490 Iasi, Romania; alin.ciobica@uaic.ro; 5 Department of Biology, Faculty of Biology, “Alexandru Ioan Cuza” University, Carol I Avenue, no 20A, 700505 Iasi, Romania

**Keywords:** SARS-CoV-2, Covid-19, safety, efficacy, vaccines

## Abstract

Severe Acute Respiratory Syndrome Coronavirus 2 is a novel strain of human beta-coronavirus that has produced over two million deaths and affected one hundred million individuals worldwide. As all the proposed drugs proved to be unstable, inducing side effects, the need to develop a vaccine crystallized in a short time. As a result, we searched the databases for articles in which the authors reported the efficacy and safety of the use of several vaccines vaccines by sex, age group, and frequency of adverse reactions. We identified a total of 19 relevant articles that were discussed throughout this manuscript. We concluded that from all eleven vaccines, three had an efficacy >90% (Pfizer–BioNTech (~95%), Moderna (~94%), and Sputnik V (~92%)) except for Oxford–AstraZeneca (~81%). However, Moderna, Sputnik V, and Oxford–AstraZeneca also alleviate severe adverse reactions, whereas in Pfizer–BioNTech this was not revealed. The remaining five (Convidicea (AD5-nCOV); Johnson & Johnson (Ad26.COV2.S); Sinopharm (BBIBP-CorV); Covaxin (BBV152), and Sinovac (CoronaVac)) were discussed based on their immunogenicity, and safety reported by the recipients since only phases 1 and 2 were conducted without clear evidence published regarding their efficacy. CoviVac and EpiVacCorona have just been approved, which is why no published article could be found. All adverse events reported following the administration of one of the four vaccines ranged from mild to moderate; limited exceptions in which the patients either developed severe forms or died, because most effects were dose-dependent. It can be concluded that aforementioned vaccines are efficient and safe, regardless of age and sex, being well-tolerated by the recipients.

## 1. Introduction

According to the World Health Organization’s (WHO) normative acts, in conjunction with the English Oxford Dictionary, a pandemic describes a "disease" whose propagation area is independent of the epicenter’s geographical localization [1].

At the end of 2019, China was severely affected by a novel strain of beta-coronavirus, having as epicenter the city of Wuhan. Without any information regarding this pathogen, the WHO declared a total pandemic several months later. Presently, over one hundred million people are positive for Severe Acute Respiratory Syndrome Coronavirus 2, and more than 2 million have died [1].

The reproduction number (R0) rate of the virus is very high: 2.24–3.58 [2] being viable on different surfaces up to 72 hours or more [3]. In this way, individuals over fifty-five years and those suffering from chronic diseases are at a higher risk, which is directly reflected by the number of deaths [4,5,6,7,8,9].

It has been demonstrated that SARS-CoV-2 binds to the angiotensin-converting enzyme 2 (ACE2) receptors [10], and systematically affects the lungs and other systems of organs. ACE2 receptors are widely expressed within distinct regions of the organism. SARS-CoV-2’s replication appeared to be dependent on the cathepsin B/L [11] and transmembrane protease, serine 2 and 4 (TMPRSS2/4) [12].

Considering how urgent the necessity of a vaccine was, we found it suitable to discuss in the present study the COVID-19 vaccines in terms of efficiency and reliability. Specifically, we will focus on the number of participants included, age group, and adverse reactions reported following the vaccination.

## 2. Methodology

Databases searched for data until February 2020 were ScienceDirect, PubMed/Medline, Scopus, and Cochrane Database of Systematic Reviews (CDSR). The searching strategy(s) employed were “AZD1222 and SARS-CoV-2”, “AZD1222 and Covid-19”, “BNT162b1 and SARS-CoV-2”, “BNT162b1 and Covid-19”, “mRNA-1273 and SARS-CoV-2”, “mRNA-1273 and Covid-19”. “rAd26 and rAd5 and SARS-CoV-2”, “rAd26 and rAd5 and Covid-19”, “AD5-nCOV and SARS-CoV-2”, “AD5-nCOV and Covid-19”, “Ad26.COV2.S and SARS-CoV-2”, “Ad26.COV2.S and Covid-19”, “BBIBP-CorV and SARS-CoV-2”, “BBIBP-CorV and Covid-19”, “BBV152 and SARS-CoV-S”, “BBV152 and Covid-19”, “CoronaVac and SARS-CoV-2”, and “CoronaVac and Covid-19”.

The adopted PubMed string was: (((AZD1222[Title/Abstract] OR BNT162b1 [Title/Abstract]) OR mRNA-1273 [Title/Abstract]) OR rAd26 and rAd5 [Title/Abstract]) OR AD5-nCOV [Title/Abstract]) OR Ad26.COV2.S [Title/Abstract]) OR BBIBP-CorV [Title/Abstract]) OR BBV152 [Title/Abstract]) OR CoronaVac [Title/Abstract]) AND SARS-CoV-2[Title/Abstract] AND COVID-19[Title/Abstract] AND humans[Title/Abstract].

Four independent authors (B.D., O.-D.I., A.C., R.M) screened the results; those that meet the eligibility criteria were further considered. Any discrepancy was solved by consent with a fifth author (C.I).

## 3. Results

### 3.1. How Efficient and Reliable COVID-19 Vaccines Are?

It should be mentioned from the beginning that we will not focused on prototypes tested until the official launch of globally distributed vaccines. The main reason why some studies were not taken into account, including those performed on experimental models, is that the current interest is focused on mass immunization in order to restrict the spread. We identified a total of 19 relevant articles in the last year that are summarized in Table 1.

As expected, studies in which more than 10,000 people soon emerged, because the scholars recommended further evidence in order to test the efficacy and safety of these vaccines.

**Table 1:** Overview regarding all studies performed until 2021 that aimed to assess the reliability and efficiency of COVID-19 vaccines.

Title of the Manuscript	Vaccine	Month and Year of Publication	Reference
Phase I/II study of COVID-19 RNA vaccine BNT162b1 in adults	BNT162b1	August 2020	[13]
Safety and Immunogenicity of Two RNA-Based Covid-19 Vaccine Candidates		October 2020	[14]
Safety and Efficacy of the BNT162b2 mRNA Covid-19 Vaccine		December 2020	[15]
An mRNA Vaccine against SARS-CoV-2—Preliminary Report	mRNA-1273	July 2020	[16]
Safety and Immunogenicity of SARS-CoV-2 mRNA-1273 Vaccine in Older Adults		September 2020	[17]
Efficacy and Safety of the mRNA-1273 SARS-CoV-2 Vaccine		December 2020	[18]
Safety and immunogenicity of the ChAdOx1 nCoV-19 vaccine against SARS-CoV-2: a preliminary report of a phase 1/2, single-blind, randomized controlled trial	AZD1222	July 2020	[19]
Safety and immunogenicity of ChAdOx1 nCoV-19 vaccine administered in a prime-boost regimen in young and old adults (COV002): a single-blind, randomized, controlled, phase 2/3 trial		December 2020	[20]
Safety and efficacy of the ChAdOx1 nCoV-19 vaccine (AZD1222) against SARS-CoV-2: an interim analysis of four randomized controlled trials in Brazil, South Africa, and the UK		December 2020	[21]
Safety and immunogenicity of an rAd26 and rAd5 vector-based heterologous prime-boost COVID-19 vaccine in two formulations: two open, non-randomized phase 1/2 studies from Russia	rAd26 and rAd5	September 2020	[22]
Safety and efficacy of an rAd26 and rAd5 vector-based heterologous prime-boost COVID-19 vaccine: an interim analysis of a randomized controlled phase 3 trial in Russia		February 2021	[23]
Safety, tolerability, and immunogenicity of a recombinant adenovirus type-5 vectored COVID-19 vaccine: a dose-escalation, open-label, non-randomized, first-in-human trial	AD5-nCOV	June 2020	[24]
Interim Results of a Phase 1–2a Trial of Ad26.COV2.S Covid-19 Vaccine	Ad26.COV2.S	January 2021	[25]
Effect of an Inactivated Vaccine Against SARS-CoV-2 on Safety and Immunogenicity Outcomes: Interim Analysis of 2 Randomized Clinical Trials	BBIBP-CorV	August 2020	[26]
Safety and immunogenicity of an inactivated SARS-CoV-2 vaccine, BBIBP-CorV: a randomized, double-blind, placebo-controlled, phase 1/2 trial		January 2021	[27]
Safety and immunogenicity of an inactivated SARS-CoV-2 vaccine, BBV152: a double-blind, randomized, phase 1 trial	BBV152	January 2021	[28]
Safety and immunogenicity of an inactivated SARS-CoV-2 vaccine, BBV152: interim results from a double-blind, randomized, multicentre, phase 2 trial, and 3-month follow-up of a double-blind, randomized phase 1 trial		March 2021	[29]
Safety, tolerability, and immunogenicity of an inactivated SARS-CoV-2 vaccine in healthy adults aged 18–59 years: a randomized, double-blind, placebo-controlled, phase 1/2 clinical trial	CoronaVac	November 2020	[30]
Safety, tolerability, and immunogenicity of an inactivated SARS-CoV-2 vaccine (CoronaVac) in healthy adults aged 60 years and older: a randomized, double-blind, placebo-controlled, phase 1/2 clinical trial		February 2021	[31]

### 3.2. mRNA Vaccines

#### 3.2.1. BNT162b1

BNT162b1 is currently, widely administered in Europe., becoming an integrated component within the current vaccination schemes. Mulligan et al. [13] were the first team that tested the vaccine manufactured by Pfizer–BioNTech in a small group of individuals. They enrolled 45 healthy adults aged between 18 and 55 years. Participants were scheduled for two doses 21 days apart—10 μg, 30 μg, or 100 μg of BNT162b1. Due to increased reactogenicity and lack of immunogenicity exhibited by the recipients after the first dose of 30 μg, the authors decided not to progress with the second dose. Local and systemic reactions were dose-dependent, varying from mild to moderate. Walsh et al. [14] deepened this research by enrolling 195 participants. They first underwent randomization—13 groups reuniting 15 participants per group. Twelve received the vaccine, while 3 received placebo. Older adults were the most susceptible group, both candidate vaccines eliciting similar dose-dependent geometric mean titers (GMT). However, the incidence of local and systemic reactions was lower in BNT162b2 than in BNT162b1, especially in older people by comparing with the 18–55 years group. At the end of last year, the study on the safety and efficacy of BNT162b2 was officially published. Polack et al. [15] reunited the largest cohort (*n* = 43.548); 43.448 received injections, from which 21.270 with BNT162b2 and 21,728 placebo. Despite the high efficiency of BNT162b2, there were situations in which participants developed COVID-19 after one week following the administration of the second dose (8 in the BNT162b2 and 162 in the placebo group). Among 10 cases of severe COVID-19, nine occurred within the placebo and one in BNT162b2 after the first dose. Noteworthy that there were no deaths, only mild to moderate side effects. Overall, this variant had an efficiency up to 95%.

#### 3.2.2. mRNA-1273

In retrospect, towards the end of July last year, the publication of the first articles on COVID-19 vaccines produced by Moderna began. The first study was that of Jackson et al. [16]. The scientists included an identical number of individuals as in Mulligan’s report. Forty-five healthy adults aged between 18 and 55 years were equally distributed into three different groups. They received two vaccinations in a dose of 25, 100, or 250 μg mRNA-1273, with a predetermined interval of 28 days apart. The antibody responses were positively correlated with the dose administered, the most common side effects promoted by mRNA-1273 including fatigue, chills, headache, myalgia, and pain to the injection site. Systemic adverse reactions were common after the second vaccination, three volunteers (21%) from the 250 μg group reporting severe adverse effects. GMT at day 29 was 40,227 in the 25-μg group, 109,209 in the 100-μg group, and 213,526 in the 250-μg group, this further suggests that the higher the dose, the higher will be the immunological response. On day 57 after the second dose, GMT were 299,751, 782,719, and 1,192,154. Another team of researchers expanded this design by including forty older adults, divided based on their age (56 to 70 and ≥71 years). Analogous, they were assigned to receive two doses of either 25 or 100 μg administered 28 days apart. The most frequently encountered adverse reactions were the same as registered in previous studies, in participants around 57 years that received 100 μg, being noted a significantly higher binding- and neutralizing-antibody titers [17]. GMT was 323,945 in individuals that had between 56 and 70 years, and 1,128,391 in elderly, respectively. Across people that received a 100-μg dose, the GMT was 1,183,066 and 3,638,522. Baden et al. [18] aimed to test the safety and efficiency of mRNA-1273 in 30.420 individuals. All participants were randomly assigned in a 1:1 ratio to receive either vaccine or placebo (*n* = 15.210 per group). According to their results, more than 96% of volunteers received both injections, whereas 2.2% had evidence of a SARS-CoV-2 infection. One hundred and ninety-six were diagnosed with COVD-19; 185 in the placebo group and 11 in the mRNA-1273. They concluded that mRNA-1273 possesses a similar efficacy across key secondary analyses, even after 14 days at assessment. Severe COVID-19 occurred in the placebo group within a small sample of 30 participants, with one fatality. The incidence was similar in the two groups, individuals displaying transient to moderate reactogenicity, serious adverse cases being rare. The overall efficiency was 94.1% in preventing COVID-19 illness.

### 3.3. Viral Vector Vaccines

#### 3.3.1. AZD1222

Compared with previously mentioned vaccines, AZD1222 is designed based on the use of viral vectors. It has been theorized on two distinct occasions [19,20] that AZD1222 might be safely used in individuals, regardless of their age. A total of 1637 were recruited and subsequently stratified based on the age-escalation manner on distinct groups (18 and >70 or older). They were assigned in a 1:1 ratio to receive either a dose of ChAdOx1 or MenACWY, but with the difference being that individuals between 56 and 70 were assigned in a different pattern: 3/5:1:3/5:1. In 21 cases, the volunteers were assigned in the non-randomized ChAdOx1 prime-boost groups (*n* = 10), did not receive the boost dose (*n* = 7), or received the incorrect vaccine (*n* = 1), while three were excluded from immunogenicity analyses because the samples were improperly labeled. The most frequently noted adverse reactions were identical with those reported by Jackson (*p* < 0.05), but more frequent in participants given ChAdOx1, that were dependent by age. The highest incidence of both local and systemic adverse reactions were noted in the 18–55 year group (*n* = 43/42), followed by 56–69 (*n* = 22/23), and 70 and older (*n* = 30/32). Even though Folegatti did not report any severe cases, Ramasamy noted thirteen during this trial. The first immune response of spike-specific T-cell reached the peak starting with week two until day 28, neutralizing antibody titers being similar across all age groups. Certain is that ChAdOx1 nCoV-19 exert an acceptable safety profile and is well-tolerated in both younger and older adults. It has a homologous boosting capacity and similar immunogenicity. Prophylactic paracetamol was indicated to diminish the severity of adverse reactions (*p* < 0.05). Chronologically, Voysey and his collaborators [21] were the first that tested AZD1222 in a cohort of 23,848 participants from which 11,636 in the interim primary efficacy analysis (7548 in the UK, 4088 in Brazil). Intriguingly, the vaccine had an overall efficacy of 62.1% in participants that received both doses given that 90.0% was the success rate in those that received only one. Vaccine’s efficacy across both groups was 70.4%, during the interval of 21 days being registered ten cases of hospitalization for COVID-19 in the control arm; two cases of severe COVID-19, and one death. There were reported a total number of 168 events of severe adverse reactions—84 in the ChAdOx1 nCoV-19 group and 91 in the control group. Moreover, three cases were classified as possibly related to a vaccine—one in the control group, one in the ChAdOx1 nCoV-19 group, and one in a participant who remained masked to group allocation.

#### 3.3.2. rAd26 and rAd5

Another viral-based vector vaccine tested is rAd26 and rAd5, in which Logunov and co-authors [22] aimed to assess the safety and immunogenicity in 67 healthy adult volunteers, aged between 18 and 60 years. In phase 1, they administered intramuscularly on day 0 one dose of rAd26-S/rAd5-S and investigated the possible beneficial activities each day for 28 days. In phase 2, vaccination began not earlier than day 5 after the first dose, being administered one dose of rAD26-S on day 0 and one of rAD5-S on day 21. Eighteen (nine per group) received either rAd26-S or rAd5-S in phase 1, whereas twenty received rAd26-S and rAd5-S in phase 2. Pain at the injection site (44 [58%], hyperthermia (38 [50%]), headache (32 [42%]), asthenia (21 [28%]), and muscle and joint pain (18 [24%]) were the most common adverse reactions that ranged from mild to moderate and with no severe adverse event. IgG titers at day 42 were 14,703 for the frozen formulation, while the neutralizing antibodies were 49.25; 11,143 with the lyophilized formulation and 45.95, with a 100% seroconversion rate. Immune responses were detected in all participants at day 28, the median cell proliferation being 2.5% CD4+ and 1.3% CD8+ for the frozen formulation, and 1.3% CD4+ and 1.1% CD8+ for that lyophilized, respectively. Afterward, Logunov et al. [23] performed another study in which they had as main objective(s) to evaluate the same parameters but at a larger scale. In this study were included a total of 21,977 aged at least 18 years. All volunteers were randomly assigned (3:1) (*n* = 16,501 vaccine/5476 placebo) to receive a dose (0.5 mL/dose) intramuscularly (rAd26) or placebo. Similar to the study of Mulligan and Voysey, participants were scheduled to receive the second dose twenty-one-day apart (rAd5). Due to the violation of the restrictions imposed on the participants, 19,866 received both doses and were included in the primary outcome analysis. Only 16 (0.1%) of 14,964 from the vaccine group and 62 (1.3%) of 4902 from placebo, respectively, were confirmed as SARS-CoV-2-positive patients. Therefore, the vaccine was efficacy in 91.6% of the cases (95% CI 85.6–95.2). The most common adverse reactions were grade 1 (7485 (94.0%) of 7966 total events. Forty-five (0.3%) of 16.427 individuals from the vaccine group and 23 (0.4%) of 5435 from the placebo exhibited severe adverse events. Unfortunately, four deaths occurred during the study (three of 16,427 in the vaccine group and one of 5435 in the placebo group). They stated that none was associated with the vaccine, the overall efficacy being 91.6%.

#### 3.3.3. AD5-nCOV

China started working on a possible vaccine to protect the population shortly after the explosion of COVID-19 worldwide. They succeeded in creating a recombinant adenovirus type-5 (Ad5) vector. As expected, they began the procedures to test its safety, tolerability, and immunogenicity. Zhu et al. [24] enrolled adults between 18 and 60 years old that were subsequently allocated to one of three intramuscular doses (5 × 1010, 1 × 1011, and 1.5 × 1011 viral particles). The safety was established on day 28, followed by a series of tests to measure the grade of neutralization and level, respectively. Even though 195 individuals were considered eligible, only 108 remained. One hundred and eight (51% male, and 49% female) were divided in three equal small groups and received low (*n* = 36), middle (*n* =36), and high (*n* = 36) doses. In 83% (*n* = 30), at least one adverse event was noted within the first week as, followed by 30 (83%) in the middle dose group, 27 (75%), and the last group. Pain at the injection site was the most common reaction in 58 (54%) of recipients, but also systemic adverse events were noted including fever (50 (46%), fatigue (47 (44%), headache (42 (39%), and muscle pain (18 (17%)). These adverse reactions varied between mild to moderate, with no serious adverse reactions 28-day post-vaccination. Humoral responses against SARS-CoV-2 reached the peak of day 28, concomitantly with a rapid specific T-cells response starting from week two.

#### 3.3.4. Ad26.COV2.S

Another candidate vaccine is Ad26, a recombinant, replication-incompetent adenovirus serotype 26 (Ad26) vector. Sadoff et al. [25] enlisted 805 participants, aged between 18 and 55 years, and over 65 years, respectively. All participants were assigned in cohort 1 and cohort 3. Long-term data regarding the efficacy of a single-dose regimen in contrast with a two-dose regimen represented cohort 2. Either a dose of 5×10^10^ viral particles or 1×10^11^ viral particles per milliliter or placebo was administered in a single or two-dose. Congruent with the aforementioned adverse reactions, fatigue, headache, myalgia, and pain-associated injection-site were prevalent. Systemic adverse reactions were less frequent in cohort 3 compared with cohort 1. Analogous observations were made for recipients of a low-dose by comparison with those who received a high-dose vaccine. Reactogenicity was low after the second dose, the neutralizing-antibody titers against wild-type virus being detected in 90% or more of all individuals on day 29 after the first dose—GMT 224 to 354. These results were independent of dose or age group, reaching 100% by day 57—GMT, 288 to 488 in cohort 1a. The antibody levels further increase and remained stable until day 71, the second dose promoting an increase by a factor of 2.6 to 2.9—GMT, 827 to 1266. Both associated antibody responses were similar, CD4+ T-cell responses being detected in 76 to 83% of the cases in cohort 1 and 60 to 67% in cohort 3. There was also noted a clear skewing toward type 1, CD8+ T-cell responses being more robust in cohort 3.

### 3.4. Conventional Inactivated Vaccines

#### 3.4.1. BBIBP-CorV

Three hundred and twenty participants were recruited, and assigned as follows: 96 to phase 1 and 224 to phase 2. Within the first phase, all 96 participants were assigned to 1 of the 3 dose groups (2.5, 5, and 10 μg/dose) and an aluminum hydroxide (alum) adjuvant-only group (*n* = 24 per group) and received 3 intramuscular injections at three distinct intervals—day 0, 28, and 56. In the phase 2, all 224 were randomized to 5 μg/dose in 2 schedule groups—day 0 and 14 (*n* = 84) versus alum only (*n* = 28), and days 0 and 21 (*n* = 84) versus alum only (*n* = 28). After one week of follow-up, the adverse reactions occurred in 3 (12.5%), 5 (20.8%), 4 (16.7%), and 6 (25.0%) patients in the alum only in phase 1, 5 (6.0%), and 4 (14.3%) in those who received injections on day 0 and 14 for vaccine and alum only, and 16 (19.0%) and 5 (17.9%) who received injections on day 0 and 21 for vaccine and alum only in phase 2, respectively. There were no severe adverse events, those commonly encountered being pain at the injection site and fever. The GMT in all three groups depending on dose after two weeks were 316, 206, and 297, and 121 and 247 after two injections on days 0 and 14, and day 0 and 21 [26]. Xia et al. [27] took further this research in an analogous manner, referring to healthy people aged between 18 and 80 years in phase 1, and phase 2 in which were included healthy adults aged 18 until 59 years. Individuals from phase 1 were divided into two groups stratified by age (18–59 years and over 60) and randomly assigned for placebo or two-dose schedule of 2 μg, 4 μg, or 8 μg on days 0 and 28. In phase 2, they were assigned in a 1:1:1 ratio, but for a single-dose schedule of 8 μg on day 0 or on a two-dose schedule of 4 μg on days 0 and 14, 0 and 21, or 0 and 28. Each cohort was subsequently stratified by a block of eight persons and allocated (3:1) to receive vaccine or placebo. At least one adverse event was noted during the follow-up at week 1 of inoculation in 42 (29%) of 144 recipients in phase 1, while in phase 2 were registered in 76 (23%) of 336 recipients (33 (39%), 8 μg day 0; 18 (21%), 4 μg days 0 and 14; 15 (18%), 4 μg days 0 and 21; and ten (12%), 4 μg days 0 and 28). One individual from the placebo group reported grade 3 fever. In terms of systemic reactions, fever was prevalent in one (4%) in the 2 μg group, one (4%) in the 4 μg group, and two (8%) in the 8 μg group; in those over 60 years, one (4%) in the 8 μg group. Fever was also dominant in (one (1%), 8 μg day 0; one (1%), 4 μg days 0 and 14; three (4%), 4 μg days 0 and 21; two (2%), 4 μg days 0 and 28). All reactions varied from mild to moderate, without any severe case at day 28 post-vaccination. Neutralizing antibody GMT were increased at day 42 in the group in which the individuals had between 18 and 59 years (87.7—2 μg group; 211.2—μg group; and 228.7—8 μg group) and over 60 years or older 80.7—2 μg; 131.5—4 μg, and 170.87—9 μg by comparison with placebo (2.0). In phase 2 cohort, the neutralizing antibodies on day 28 were significantly higher in the 4 μg days 0 and 14 (169.5), days 0 and 21 (282.7), and days 0 and 28 (218.0) schedules than the 8 μg day 0 (65.4–99.6), 2 μg group; 131.5 (108.2–159.7), 4 μg group; and 170.87 (133.0–219.5), 8 μg schedule (14.7; all *p* < 0.001).

#### 3.4.2. BBV152

Similar to other clinical trials, Ella and co-authors had as main objective to investigate the efficiency of a whole-virion inactivated vaccine in 19 hospitals across India [28,29]. Although 1748 individuals were recruited, only 755 (43.19%—aged between 12 and 65 years) met the eligibility criteria. Eight hundred and twenty-seven volunteers were screened, but only three hundred and seventy-five met the eligibility criteria. Volunteers were randomly assigned in a 1:1 ratio to receive (3 µg with Algel-IMDG, 6 µg with Algel-IMDG, or 6 µg with Algel) or an Algel only control. Two intramuscular doses were administered on day 0, and day 14/28 depending on design. In contrast with phase 1 in which both local and systemic were reported in 62 (*n* = 17; 17% in 3 μg with Algel-IMDG group, 21—21% in the 6 μg with Algel-IMDG group, 14—14% in the 6 μg with Algel group, and ten—10% in the Algel-only group), in phase 2 there were no significant difference among groups (*n* = 38—20% in 3 µg with Algel-IMDG of 190 and *n* = 40—21.1% in the 6 µg with Algel-IMDG of 190) in days 0–7 and 28–35. Pain at the injection site (17 (5%)), headache (13 (3%)), fatigue (11 (3%)), fever (nine (2%)), and nausea or vomiting (seven (2%)) were prevalent; mild (43 (69%) of 62) or moderate (19 (31%)) were also frequent after the first dose, with only one severe case reported in the 6 μg with Algel group. Both CD4+ and CD8+ T-cell were detected in a subset of recipients in both Algel-IMDG groups, the seroconversion rates being 87.9, 91.9, and 82.8 in the 3 μg with Algel-IMDG, 6 μg with Algel-IMDG, and 6 μg with Algel groups. Seroconversion based on MNT50 at day 56 was reported in 162 (88.0% of 184 participants) in the 3 μg with Algel-IMDG group and 171 (96.6% of 177 participants) in the 6 μg with Algel-IMDG group. GMT and plaque-reduction neutralization test (PRNT_50_) at day 56 were higher in the 6 μg with Algel-IMDG group (197.0) than the 3 μg with Algel-IMDG group (100.9; *p* = 0.0041). Seroconversion based on PRNT_50_ at day 56 was observed in 171 (92.9% of 184 participants) in the 3 μg with Algel-IMDG group and 174 (98.3% of 177 participants) in the 6 μg with Algel-IMDG group. GMTs (MNT50) at day 56 were 92.5 in the 3 μg with Algel-IMDG group and 160.1 in the 6 μg with Algel-IMDG group. The 3 μg with Algel-IMDG and 6 μg with Algel-IMDG formulations eliciting T-cell responses were biased to a Th1 phenotype at day 42. From the phase 1 trial, 3-month post-second-dose GMTs (MNT50) were 39.9 in the 3 μg with Algel-IMDG group, 69.5 in the 6 μg with Algel-IMDG group, 53.3 in the 6 μg with Algel group, and 20.7 in the Algel alone group.

#### 3.4.3. CoronaVac

Another Chinese vaccine that has been recently approved and released is CoronaVac, an inactivated vaccine candidate [30,31]. A total of 1166 participants were enrolled in phase 1 and phase 2 and assigned in a 1:1/2:2:2:1 ratio; (n = 72/144), and (*n* = 600/350). The incidence of adverse reactions between day 0 and 14 was 29% (*n* = 7) in the 3 μg group, 38% (*n* = 9) in the 6 μg group, and 8% (*n* = 8) in the placebo of 24 individuals per block, whereas between day 0 and 28 was 13% (*n* = 3) in the 3 μg, 17% (*n* = 4) in the 6 μg and 13% (*n* = 3) in the placebo of 24/23 participants, respectively. Interestingly, Wu reported a higher incidence of adverse events was 20% (*n* = 20) in the 1.5 μg of 100 individuals, 20% (*n* = 25) in the 3 μg of 125 individuals, 22% (*n* = 27) in the 6 μg of 123 individuals, and 21% (*n* = 21) of 73 in the placebo after 28 days. Pain at the injection site (*n* = 39/9% of 421 individuals) was commonly encountered, with seven severe adverse cases unrelated to vaccination (2%). In phase 1, the seroconversion on day 0 and 14 was noted in 46% (*n* = 11) in the 3 μg from 24 people, 50% (*n* = 12) in the 6 μg from 24 people, and 0% in the placebo from 24 people, whereas on day 28 was 83% (*n* =20) in the 3 μg, 79% (*n* = 19), and 4% (*n* = 1) in the placebo from 24 individuals. Thankfully, potent results were obtained in the second study when a 100% seroconversion was noted in the 3 μg group from 24 individuals, followed by a 95.7% (*n* = 22 of 23) in the 6 μg group. The seroconversion in phase 2 was 90.7% (*n* = 88 of 97) in the 1.5 μg group, 98.0% (*n* = 96 of 98) in the 3 μg, and 99.0% (*n* = 97 of 98) in the 6 μg. There were no detectable antibody response in placebo, of neutralizing antibodies being 92% (*n* = 109) in the 3 μg of 118 individuals, 98% (*n* = 117) in the 6 μg from 119, and 3% (*n* = 2) in the placebo from 60 volunteers at day 0 and 14. At day 28 was 97% (*n* = 114) from 117 in the 3 μg, 100% in the 6 μg from 118, and 0% from 59 in the placebo.

## 4. Conclusions

Based on aspects discussed throughout this manuscript, we concluded that not all vaccines manufactured are safe. Nevertheless, the risk of severe adverse events or even death was low. Cumulatively, over 100,000 individuals took part in these studies, both common local and systemic adverse reactions being dose-dependent, and ranging from mild to moderate and based on the age of the individuals (Table 2). We also found it suitable to summarize in Table 2 the efficiency of these vaccines in diminishing both mild and moderate to severe cases. They are organized depending on technique used to synthesize these vaccines. Unfortunately, in only four situations the authors reported the efficacy of the respective vaccines. We considered information on the effectiveness of vaccines that were not found in published articles to be inadequate. Analogous for the two Russian vaccines, CoviVac (https://clinicaltrials.gov/ct2/show/NCT04619628, accessed on 12 March 2021) and EpiVacCorona (https://clinicaltrials.gov/ct2/show/results/NCT04527575, accessed on 4 May 2012) (https://clinicaltrials.gov/ct2/show/NCT04780035, accessed on 12 March 2021), which have just been accepted and the trial is underway.

**Table 2:** The efficiency of all vaccines on both mild to moderate and severe adverse reactions based on all available literature.

Vaccine	Mild to Moderate and Severe Adverse Reactions	Trial Location
Pfizer–BioNTech (BNT162b1)	~95%/Not reported	Multinational
Moderna (mRNA-1273)	~94%/~100%	Unites States
Sputnik V (rAd26 and rAd5)	~92%/~100%	Russia
Oxford–AstraZeneca (AZD1222) [32]	~81%/~100%	Multinational
Convidicea (AD5-nCOV)	Not reported	Not reported
Johnson & Johnson (Ad26.COV2.S)	Not reported	Not reported
Sinopharm (BBIBP-CorV)	Not reported	Not reported
Covaxin (BBV152)	Not reported	Not reported
Sinovac (CoronaVac)	Not reported	Not reported

Despite all the evidence, several questions remained without an answer: (1) Possible long-term side effects; (2) Vaccination of pregnant or that become pregnant in the assigned interval between the first and second doses, respectively; (3) The hypothesis that variant B117 discovered in the UK is more virulent for children; (4) If the method of detecting the presence of SARS-CoV-2 remains the same, how viable the vaccines will be as long as mutations have been identified; nine strains with mutations within BN501Y, E484K, and L452R; (5) and Should the current vaccines be optimized following the discovery of these mutations.

## Data Availability

The datasets used and analyzed during the current study are available from the corresponding author upon reasonable request.
